# From trial data to personalized medicine: a validated framework with an application to Crohn’s disease

**DOI:** 10.1038/s41746-025-01627-w

**Published:** 2025-05-31

**Authors:** Vivek A. Rudrapatna, Vignesh G. Ravindranath, Douglas V. Arneson, Arman Mosenia, Atul J. Butte, Shan Wang

**Affiliations:** 1 Division of Gastroenterology, Department of Medicine, University of California, San Francisco, CA USA; 2 Bakar Computational Health Sciences Institute, University of California, San Francisco, CA USA; 3 School of Medicine, University of California, San Francisco, CA USA; 4 Department of Mathematics and Statistics, University of San Francisco, San Francisco, CA USA

**Keywords:** Outcomes research, Health care, Inflammatory bowel disease

## Abstract

Clinical practice is currently guided by studies that average over patient outcomes. This may not be the best approach, as different patients may have different treatment responses. Here we extend a method for simulating clinical trials to identify optimal treatments for each patient, and we illustrate this approach in the context of Crohn’s disease. Using the data from 15 randomized trials (*N* = 5703), we used statistical hypothesis testing to identify seven subgroups with distinct responses to three different drug classes. The largest subgroup consisted of patients with equivocal responses to all drug classes, whereas the second largest showed superiority with anti-TNFs. We also identified a subgroup of women over 50 with superior responses to anti-IL-12/23s. Interestingly, this group appeared under-represented in the trials (2%) compared to patients at the University of California (25%). Overall, these results underscore the importance of studying personalized medicine, demonstrate the value of clinical trial data, and provide a roadmap for applying this method broadly across diseases. These results also highlight the importance of diverse and representative recruitment into clinical trials.

## Introduction

Clinical practice today is guided by randomized controlled trials (RCTs), the gold-standard for studying medical interventions. Nonetheless, RCTs have their limitations. For one, while they are typically used to learn which interventions are the best *on average* in study cohorts, they are generally not designed to identify the best intervention for any given individual. This is a problem whenever there is significant heterogeneity across patients, as is common in most diseases.

The concepts of personalized and precision medicine have emerged in recent years as a counterpoint to the current strategy of treating individuals based on the results of cohort-averaging studies. However, methods for robustly learning optimal personalized treatments have been sparse, and concrete illustrations using real clinical data are even more rare. The needs for analytical innovation and validation in personalized medicine are especially acute, given 1) the growing expense of conducting RCTs and 2) the even greater infeasibility of conducting many RCTs in many homogenous subgroups, or in the limit, in individual patients.

The importance of learning optimal personalized treatments is well illustrated by Crohn’s disease (CD), a gastrointestinal disorder characterized by diverse phenotypes and treatment responses. Over the last two decades many drugs have been approved for Crohn’s disease based on placebo-controlled RCTs. What has generally been unclear is how well these drugs perform against each other on average, much less how well they perform in any one patient. Answering the first of these would ideally require head-to-head RCTs, but their significant expense has resulted in a large evidence void.

Network meta-analyses (NMAs) have been prioritized to address these gaps, using summary statistics from historical trials to infer relative effectiveness. A recent NMA in CD found anti-tumor necrosis factor alpha (anti-TNF) drugs to be most effective at inducing remission, followed by anti-interleukin-12/23s (anti-IL-12/23s) and anti-integrins^1^. However, NMAs have generally not been validated as being predictive of future RCTs, and require many strong assumptions that do not hold in practice.

Individual participant data meta-analyses (IPDMAs) are the gold standard for meta-analyses^2,3^ and offer a unique opportunity to account for patient heterogeneity and discover subgroups with different treatment responses. More importantly, this approach can be used to learn true causal effects. In a recent IPDMA^4^, we developed and validated a new method for using historical RCT data to predict the results of future head-to-head trials. We showed that this method (sequential regression and simulation; SRS) works even in the presence of significant inter-trial heterogeneity, and validated its use by correctly predicting the results of SEAVUE^5^, a recent trial of adalimumab versus ustekinumab for CD.

Here we extend this validated approach to its logical next step on the road to personalized medicine. Briefly, we used SRS to simulate many head-to-head trials on virtual cohorts of digital twins, and we looked for differences in potential efficacy. We used these findings to motivate a broader assessment of how personalized treatment choices can lead to better outcomes compared to general rules.

## Results

### Cohort characteristics

**Fig. 1:**
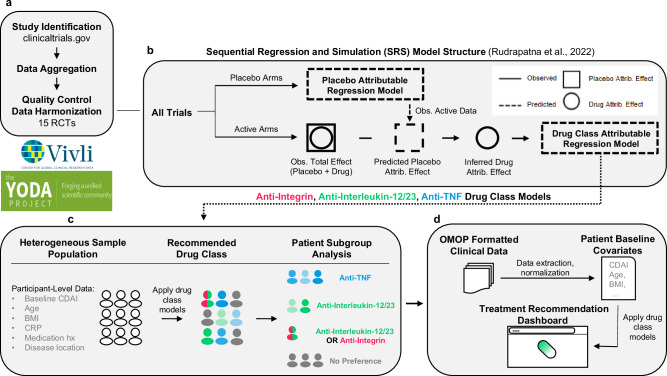
Overview. **a** Clinical trials were found using clinicaltrials.gov and sought for retrieval on the YODA and Vivli platforms. Individual participant data (IPD) from trials that collected CDAI scores at week 6 visits were then aggregated and harmonized. **b** Using sequential regression and simulation, a method for normalizing clinical trial data against a common placebo rate, a placebo-attributable model and three drug-attributable models - anti-integrin, anti-interleukin-12/23 and anti-TNF - were developed. Disease activity reduction was partitioned into placebo-attributable (square) and drug-attributable (circle) effects based on baseline covariates (age, sex, BMI, etc.). IPD (solid lines) were used to predict or simulate data (dashed lines). **c** The drug-attributable models were utilized to simulate patient-level outcomes post-treatment (counterfactuals). Pairwise t-tests (*p* < 0.05) were conducted to compare and rank the mean responses for all drug classes - anti-integrin vs anti-interleukin-12/23, anti-integrin vs anti-TNF, and anti-interleukin-12/23 vs anti-TNF - and assign patients into one of seven subgroup memberships (see Table 3). **d** Lastly, the models were re-packaged into a prototype decision support tool that uses manual inputs and optionally, OMOP-formatted data, to recommend treatments for individual patients.

See Fig. 1 for an overview of this study. Our cohort consisted of 5703 participants, drawn from fifteen trials of all FDA-approved biologics as of 2019^6–19^ (Supplementary Information). These biologics corresponded to three drug classes: anti-TNFs, anti-IL-12/23 s, and anti-integrins. The members of our cohort were generally similar in their univariate characteristics across trials and eligibility criteria (Table 1; Supplementary Table 1). One exception to this pertained to the history of anti-TNF exposure, which affected 16%, 56%, and 74%, of the anti-TNF, anti-Integrin, and anti-IL-12/23 cohorts respectively. This variable, and all other variables listed in Table 1, were used in regression models to control potential biases that otherwise could result from a naïve pooling of cohorts.

**Table 1:** Baseline characteristics of the meta-analyzed study cohort, stratified by trial

Drug Class Trial Alias	Anti-Il-12/23			Anti-Integrin			Anti-TNF								
	CERTIFI	UNITI1	UNITI2	ENACT	GEMINI2	GEMINI3	ACCENT	CLASSIC	EXTEND				PRECISE1	PRECISE2	SONIC
Trial NCT identifier	**NCT00771667**	**NCT01369329**	**NCT01369342**	**NCT00032786**	**NCT00783692**	**NCT01224171**	**NCT00207662**	**NCT00055523** **NCT00055497**	**NCT00348283**	**NCT00291668**	**NCT00552058**	**NCT02499783**	**NCT00152490**	**NCT00152490**	**NCT00152425**
Year	**2008**	**2011**	**2011**	**2001**	**2008**	**2010**	**1999**	**2002**	**2006**	**2006**	**2008**	**2015**	**2003**	**2003**	**2005**
Included cohort size	**252**	**502**	**416**	**875**	**1050**	**407**	**190**	**73**	**64**	**60**	**409**	**102**	**593**	**559**	**151**
**Treatment Group**															
Active	126 (50%)	252 (50%)	209 (50%)	698 (80%)	915 (87%)	205 (50%)	190 (100%)	73 (100%)	64 (100%)	30 (50%)	210 (51%)	102 (100%)	298 (50%)	559 (10%)	151 (100%)
Placebo	126 (50%)	250 (50%)	207 (50%)	177 (20%)	135 (13%)	202 (50%)	..	..	..	30 (50%)	199 (49%)	..	295 (50%)	..	..
**Age**	39 ( ± 13)	37 ( ± 12)	39 ( ± 13)	38 ( ± 13)	36 ( ± 12)	38 ( ± 13)	37 ( ± 12)	38 ( ± 11)	37 ( ± 11)	33 ( ± 12)	37 ( ± 12)	33 ( ± 10)	37 ( ± 12)	39 ( ± 13)	36 ( ± 13)
**Female Sex**	107 (42%)	224 (45%)	189 (45%)	372 (43%)	492 (47%)	176 (43%)	79 (42%)	35 (48%)	24 (38%)	47 (78%)	181 (44%)	67 (66%)	259 (44%)	271 (48%)	77 (51%)
**BMI**	26 ( ± 7.3)	22 ( ± 0.58)	25 ( ± 6.2)	25 ( ± 5.6)	24 ( ± 6.0)	24 ( ± 5.7)	24 ( ± 4.7)	26 ( ± 6.0)	25 ( ± 4.6)	20 ( ± 2.7)	24 ( ± 4.9)	19 ( ± 2.7)	24 ( ± 5.3)	24 ( ± 5.1)	25 ( ± 5.5)
**Baseline CDAI**	320 ( ± 67)	320 ( ± 60)	300 ( ± 56)	300 ( ± 61)	320 ( ± 69)	310 ( ± 53)	310 ( ± 54)	290 ( ± 52)	320 ( ± 69)	290 ( ± 58)	290 ( ± 60)	270 ( ± 48)	300 ( ± 61)	300 ( ± 59)	290 ( ± 62)
**CRP (mg/L)**	21 ( ± 28)	18 ( ± 23)	16 ( ± 20)	19 ( ± 25)	21 ( ± 26)	18 ( ± 22)	20 ( ± 23)	13 ( ± 18)	20 ( ± 21)	31 ( ± 21)	19 ( ± 26)	24 ( ± 25)	18 ( ± 25)	20 ( ± 28)	25 ( ± 28)
**History of Anti-TNF Use**	252 (100%)	479 (95%)	135 (32%)	348 (40%)	649 (62%)	306 (75%)	0 (0%)	2 (3%)	31 (48%)	1 (2%)	0 (0%)	0 (0%)	161 (27%)	156 (28%)	0 (0%)
**Steroid Use**	134 (53%)	232 (46%)	172 (41%)	356 (41%)	532 (51%)	213 (52%)	94 (49%)	21 (29%)	6 (9%)	14 (23%)	179 (44%)	31 (30%)	235 (40%)	205 (37%)	58 (38%)
**Imm. use**	64 (25%)	166 (33%)	143 (34%)	330 (38%)	341 (32%)	137 (34%)	44 (23%)	19 (26%)	26 (41%)	17 (28%)	139 (34%)	61 (60%)	235 (40%)	230 (41%)	0 (0%)
**Ileal Disease**	182 (72%)	407 (81%)	335 (81%)	675 (77%)	752 (72%)	310 (76%)	155 (82%)	47 (64%)	48 (75%)	42 (70%)	286 (70%)	82 (80%)	425 (72%)	378 (68%)	104 (69%)

Characterization of baseline covariates of included studies. Placebo arms from ACCENT, CLASSIC, EXTEND, NCT02499783, PRECISE2, and SONIC studies were not included due to the absence of a 6-week parallel arm placebo group (see Supplementary Fig. 1). Continuous variables are reported as mean (standard deviation) and binary variables are reported as count (proportion). CRP c-reactive protein, TNF tumor necrosis factor.

### Placebo model

To address the potential bias that could result from a naive pooling of subjects across trials, we used sequential regression and simulation (SRS) to normalize the data and analytically separate the drug-attributable component of the patient response from the placebo effect^4^. SRS uses nested linear mixed-effects models to estimate the association between baseline covariates and outcomes. Prior applications of SRS to Crohn’s disease have shown that this model class performs similarly to that of non-parametric machine learning models^4^.

We began by modeling the placebo response, using the subset of the participants who were assigned to receive placebo (*N* = 1621). We modeled their week 6 response as a function of all captured covariates and study year (fixed effects) as well as trial of origin (random effect). This model was highly significant (*p* < 0.001; Table 2), consistent with the placebo effect being at least partially predictable.

**Table 2:** Linear mixed effect regression models of the reduction in CDAI at week 6

	Placebo			Anti-IL-12/23			Anti-Integrin			Anti-TNF		
**Predictors**	*Estimate*	*Std. Error*	*p-value*	*Estimate*	*Std. Error*	*p-value*	*Estimate*	*Std. Error*	*p-value*	*Estimate*	*Std. Error*	*p-value*
**Intercept**	74.69	9.48	**<0.001**	22.19	22.01	0.356	36.82	7.34	**<0.001**	54.96	9.80	**<0.001**
**Year (Centered)**	−1.96	0.99	0.096	..	..	..	..	..	..	..	..	..
**Baseline CDAI (Centered)**	0.33	0.04	**<0.001**	−0.03	0.06	0.640	0.02	0.04	0.590	0.11	0.04	**0.002**
**Age (Centered)**	0.41	0.18	**0.025**	0.30	0.30	0.313	−0.54	0.19	**0.004**	−1.23	0.18	**<0.001**
**BMI (Centered)**	0.72	0.43	0.098	−1.69	0.75	**0.024**	−0.35	0.39	0.380	−0.19	0.44	0.660
**CRP (mg/L) (Centered)**	−0.22	0.10	**0.022**	0.48	0.16	**0.002**	0.12	0.09	0.196	0.35	0.08	**<0.001**
**History of Anti-TNF Use**	−27.31	5.24	**<0.001**	28.00	12.06	**0.021**	1.66	4.49	0.712	7.03	6.09	0.249
**Sex: Male**	2.22	4.42	0.616	−6.41	7.74	0.408	-4.03	4.32	0.351	0.68	4.16	0.871
**Steroid Use**	0.32	4.44	0.943	19.82	7.60	**0.009**	4.97	4.32	0.251	−1.69	4.30	0.694
**Immunomod. Use**	−1.74	4.69	0.711	0.54	8.28	0.948	−4.35	4.54	0.338	−1.70	4.55	0.708
**Ileal Disease**	4.88	5.05	0.333	−9.14	9.64	0.344	−8.15	4.97	0.102	−7.29	4.58	0.111

We fit a total of four linear mixed-effects regression models: one placebo model and three nested models of the drug class-attributable response. Rows correspond to the fixed effect parameters of each model, and columns correspond to the estimated coefficients, standard errors, and Wald test *p*-values with bolding corresponding to significance at the 0.05 level. Year was not used for the drug class models due to insufficient variation (few trials per drug class, clustered together in calendar time).

We identified six statistically significant predictors. The coefficient for the study year was negative, suggesting a reduction in measured placebo effects over time. History of anti-TNF use was associated with 27 points less of a placebo effect, consistent with prior studies. Baseline CDAI was also a significant predictor: every 100 points of a higher baseline CDAI (restricted by trial eligibility criteria to fall between 220 and 450) was associated with 33 points more spontaneous improvement after 6 weeks. This was consistent with regression to the mean. Age and c-reactive protein (CRP) were also significant albeit with small effects. Most of the explainable variation in the placebo effect was accounted for by these explicitly captured clinical factors and study year; only 1% of the total variation was attributable to other non-specific heterogeneity across the included trials.

### Drug class models

We used the placebo model to calculate the mean placebo-attributable response for each participant assigned to receive active treatment (*N* = 4082) and subtract this from their observed response, leaving behind the drug-attributable reduction in CDAI. We then used the residuals to fit three additional mixed effects models, one per drug class.

The drug class models were significant (*p* < 0.01 for all; Table 2). We identified ten predictors across drug classes. Efficacious responses to IL12/23s were positively associated history of anti-TNF use (28 additional points of CDAI reduction) and steroid use (20 additional points). Elevated CRP was associated with a positive response to IL12/23 s, whereas elevated body mass index (BMI) was associated with a negative response. For the anti-integrin class, each decade of life was associated with 5 points less of a response on the CDAI. Lastly, for the anti-TNF class, we identified three additional predictors of efficacy beyond the intercept term. Elevations in baseline CDAI and CRP were associated with increased efficacy, whereas age was inversely associated (12 points less of CDAI reduction for each decade).

To help improve the efficiency of future trials, we compared significant coefficients identified in the placebo and active treatment models. Five coefficients had opposite effects: age, BMI, CRP, history of anti-TNF use, and ileal involvement (Table 2). These results implied that young patients with lower BMIs, no prior anti-TNF use, elevated CRP, and colonic disease would be expected to have the widest margin of difference between placebo and treatment arms; thus, trials performed in this group would be expected to have the greatest statistical power to detect evidence of efficacy. This result underscored the value of separating placebo- and drug-attributable effects using separate regression models; a regression model lacking these implied interaction terms would have missed these findings.

### Subgroups

We simulated potential outcomes for all participants under each drug class and performed pairwise t-tests to rank-order treatment preferences and define subgroups. We identified seven subgroups (Table 3). The largest subgroup (55%, *N* = 3142) consisted of patients who didn’t appear to have selective efficacy with any one drug class. The next largest group showed evidence for an anti-TNF being best or tied-for-best (42%, *N* = 2418). These results explain prior findings favouring anti-TNFs as being the result of using “majority vote” statistical methods in a situation where most participants “abstain”.

**Table 3:** Treatment subgroups

Drug Class Preference	Subgroup	*N* (%)
Anti-TNF	TNF > (IL = INT)	2021 (35)
	TNF > INT > IL	43 (0.8)
Anti-TNF, Anti-Interleukin-12/23	(IL = TNF) > INT	354 (6)
Anti-Interleukin-12/23	IL > (TNF = INT)	138 (2.5)
	IL > TNF > INT	1 (0.02)
Other	(TNF = INT) > IL	4 (0.07)
No Preference	(TNF = IL = INT)	3142 (55)

The finalized mixed effects models were used to simulate counterfactual outcomes under all possible treatment scenarios. The modeled outcomes and the associated uncertainties in these outcomes were used to perform pairwise t-testing to assess evidence for rank-ordered preferences across drug classes. Distinct patterns of rank-orderings were used to establish membership in one of 6 subgroups. Subjects without sufficient statistical evidence (alpha = 0.05) of a more efficacious response to any one drug class were placed into a 7th category (no preference). TNF = anti-tumor necrosis factor, IL = anti-interleukin-12/23, INT = anti-integrin.

**Fig. 2:**
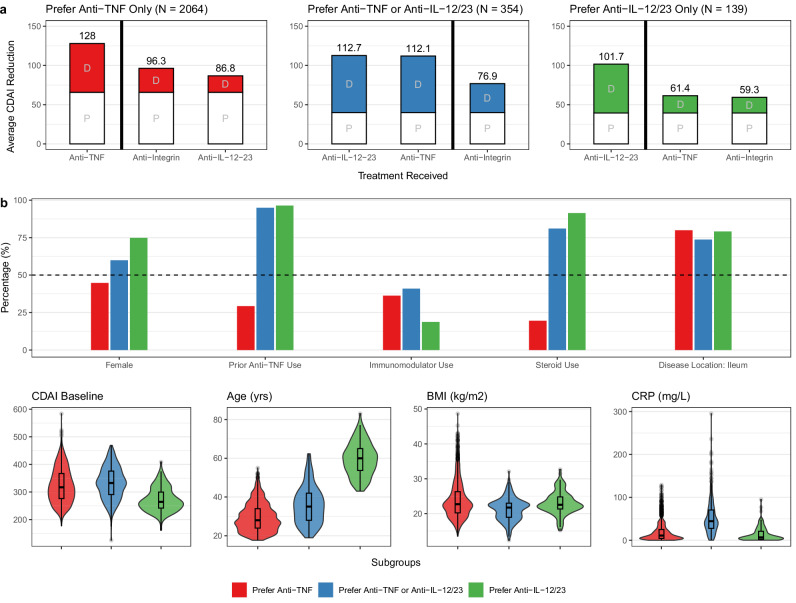
Subgroup analysis. Detailed comparison of three major subgroup cohorts found in the trial-based cohort (*N* = 5703): prefer anti-TNF only (*N* = 2061, red), prefer anti-TNF or anti-IL-12/23 (*N* = 355, blue), and prefer anti-IL-12/23 only (*N* = 139, green). **a** Estimated treatment effects by subgroups. The bar plots on the left show the average placebo (P) and drug-class (D) attributable effects for each subgroup. Superior drug classes (left of bolded vertical line) reduce disease activity (CDAI reduction) by 30-40 points more on average compared to non-superior drug classes (right of bolded vertical line). **b** Covariate distributions by subgroup.

We also identified a subgroup whose responses deviated from the majority, and thus might be harmed by “one-size-fits-all” treatment guidelines that are informed by cohort-averaging studies. Specifically, we identified a subgroup of 139 patients who showed superior efficacy with an anti-IL-12/23, achieving 40 points greater reduction in the CDAI compared to the other drug classes (Fig. 2). 50% of these patients were predicted as achieving clinical response (CDAI reduction of 100 points or more) at week 6, compared to only 3% with an anti-TNF. This subgroup was predominantly female, over the age of 50, had a history of anti-TNF exposure, had relatively lower CDAIs at baseline, and were receiving steroids (Supplementary Table 2).

Given the small size of this subgroup, and the general risk of overfitting and false discoveries due to multiple hypothesis testing, we repeated the analysis using 10-fold cross-validation. In each fold, we used 90% of the data to estimate parameters for the placebo and drug class models, and used these models to assign held-out patients to subgroups using pairwise t-testing. This analysis consistently found this anti-IL-12/23 subgroup across all ten folds, and consistently found a demographic association between this subgroup and women over 50 (Supplementary Tables 3, 4).

The anti-IL-12/23 subgroup corresponded to only 2% of the overall trial population. Given this, we wondered if a decision support tool that selectively recommends this drug class would have any measurable value in clinical practice. Thus, we queried the University of California Health Data Warehouse, a multicentre database of health records data, to identify patients who might belong to this subgroup and thus could benefit from a personalized treatment recommendation tool. We found that 25% of the patients seen for Crohn’s disease were women over the age of 50 (*N* = 5647; 2012-2022) (Supplementary Fig. 5). This striking difference in cohort prevalence (25% at the University of California vs 2% in the trials) suggested the possibility of implicit selection bias in these trials. Supporting this view, we found Black participants to be significantly underrepresented (2% in the trials; Supplementary Table 5).

When limiting our queries to the timeframe when all drug classes were FDA-approved, we noted that 75% of biologic-exposed women over 50 did not receive an anti-IL12/23 as their next treatment. This suggested a potential future role for software-aided treatment optimization.

Since the existence of this anti-IL-12/23-preferring subgroup was a new and potentially testable hypothesis raised by this analysis, we performed a sample size calculation to determine the feasibility of further validation via a prospective study. We calculated that a trial with 250 participants in each arm would have 87% power to show superiority of anti-IL-12/23 s over anti-TNFs in all patients over the age of 50. If further restricted to just women over 50, this potential trial was calculated as having 97% power (Supplementary Table 6).

### Decision support

**Fig. 3:**
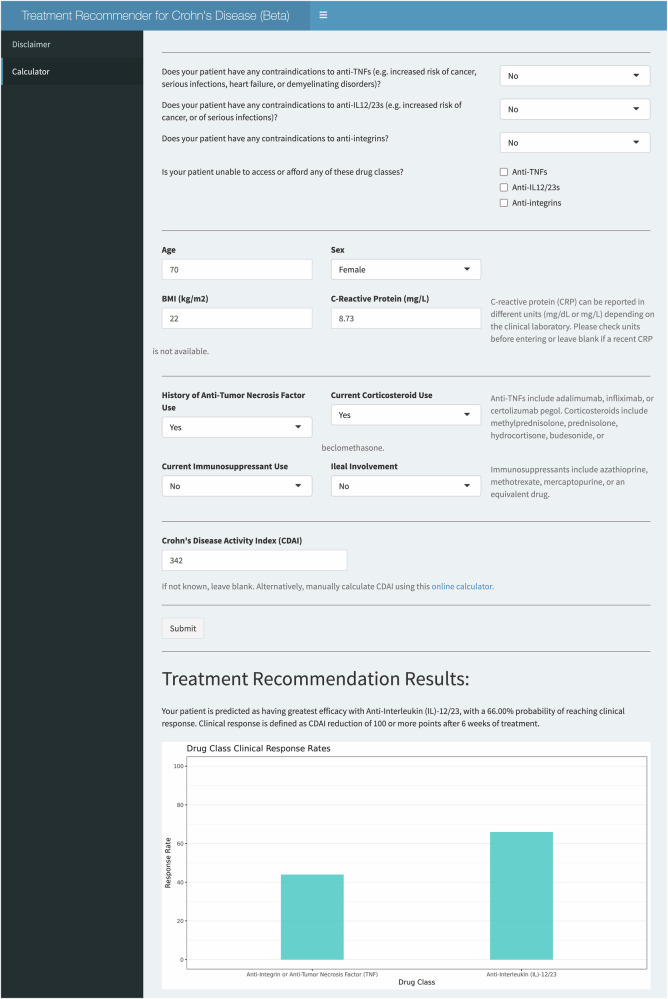
Treatment recommendation dashboard. Example user interface and output of R Shiny treatment recommendation dashboard.

To bridge these findings to the clinic we have prototyped a decision support tool (crohnsrx.org). It uses manual inputs on patient-level features to produce treatment recommendations (Fig. 3). We have provided additional guidance to help clinicians interpret the output and avoid incorrectly using the tool. Examples of incorrect use include: 1) applying the tool to patients who do not resemble the subjects used to train the model (e.g., don’t retrospectively meet trial eligibility criteria), and 2) using the tool to decide whether to start combination therapies (e.g., immunomodulators + biologics). The latter was out of scope for this study due to insufficient trial data to estimate the effect of combination therapies. Guidance on the website clarifies that immunomodulators are an input in the model, but they are used to calculate treatment outcomes when these choices have *already been made* by the clinician prior to using the model (rather than being recommended by the model).

### Sensitivity analyses

Our chosen modeling approach used linear mixed effects models. This model class has many advantages, including 1) quick inference time (important for feasible use in clinical practice), 2) interpretability, and 3) ability to perform post-hoc statistical hypothesis testing (currently not well developed for machine learning). However, our models lack interactions and non-linearities, features that could be important in identifying a patient’s optimal subgroup.

Thus we sought to test the sensitivity of our subgroup analysis to the choice of model family. In prior work using this dataset^4^, we evaluated several model classes and found random forests (RFs) to be the most accurate at predicting placebo responses, slightly better than the linear mixed model (cross-validated RMSE of 92.9, vs 93.2). RFs naturally encode interactions and non-linearities, and thus we repeated the complete subgroup assignment procedure using this class instead. We found that that the linear model was 92% concordant in subgroup assignment relative to the RF model. This high degree of concordance was reassuring, although it also implies that interactions and non-linearities likely have a role in predicting treatment responses in Crohn’s disease.

As a final sensitivity analysis, we considered the possibility that users of our tool may not have all inputs available, and asked to what extent our method would produce contradictory subgroup assignments. For example, recommending drug class B over A, when the original subgroup implies that A is superior to B. We individually ablated three predictors felt most likely to be missing in clinical practice: baseline CDAI, CRP, and ileal involvement. On these assessments, we found that the concordance rate was 100% for each of these three ablation experiments, relative to the full model. While these reduced models occasionally had less power to resolve differences seen in the full model, they never identified an ordinal relationship that contradicted the full model.

## Discussion

Here we demonstrate a new method for using historical RCT data to learn the best treatment choices for individual patients. Although we illustrate this concept specifically in the context of Crohn’s disease and on the outcome of efficacy, the approach is generalizable and applies to other diseases and other outcome measures like safety. Our approach demonstrates the tremendous value contained within historical RCT data, data that was previously inaccessible but is now generally available for secondary re-use. Our illustration of Crohn’s disease concretely demonstrates how widespread the heterogeneity in treatment effects appear to be in this disease and probably many others. It also illustrates the potential harms of “one-size fits all” clinical evidence, as well as the distortions that can result from biased recruitment into RCTs. Overall this work offers an important tool to researchers in the fields of personalized and precision medicine.

Our study also uncovers many findings of basic and practical significance to the CD research community, starting with the result that the wide variability in observed responses to CD treatments is partially predictable from standardly captured features. Other implications of our study include: 1) some women over 50 should likely receive anti-IL-12/23s s line after failing to respond to an anti-TNF and, 2) many patients may not have superior induction efficacy with any of the three drug classes studied here, irrespective of prior-TNF exposure, and other treatment selection criteria should be used (safety, tolerability, cost). We did not identify any subgroups who should be recommended anti-IL12-23s first-line, nor did we find any subgroups with preferential efficacy to anti-Integrins. Rather than proposing specific revisions to clinical guidelines and decision trees, our study generally implies that integrated decision support tools can help clinicians select better treatments for individual patients. Other notable findings include 1) evidence of implicit selection bias impacting registrational trials, and 2) combinations of features that predict placebo and drug-class responses, which could be used to design more efficient clinical trials in the future.

Our work builds on previous evidence synthesis efforts in CD, particularly NMAs^1,20^. Recent NMAs have found that anti-TNF drugs appear to be the most efficacious drug for inducing clinical remission, irrespective of prior biologic exposure^1^. Although we used a similar set of trials as that study, we came to a slightly different conclusion: most of the subjects in these trials *do not* appear to preferentially benefit from any of three currently approved drug classes. Instead, we found that patients favouring anti-TNFs were actually in the minority, albeit a large one (42%). This apparent contradiction can be understood as the result of an “ecological fallacy”, where one incorrectly deduces that a cohort-averaged effect also applies to each member of the cohort. An apt analogy would be of an election where the majority abstains, and the next largest constituency “votes” for an anti-TNF.

Thus, our findings are in fact consistent with prior NMAs that instead rely on aggregate statistics from trials. However, these findings more generally suggest that the field of evidence synthesis must increasingly embrace IPD to generate results that are more precise and less susceptible to misinterpretation. Methods such as SRS can add additional credibility and reduce the dependence on strong homogeneity assumptions implicit in traditional pooled analyses of IPD. This method also enables deeper insights into the overall patient response as the result of two distinguishable effects: placebo-attributable and drug-attributable. The predictors of the placebo effect that we identified here were consistent with the prior literature^21^. Yet we identified more predictors than have previously been reported, likely because our method implicitly accounts for drug-by-effect interactions that are often unmodeled in one-step IPD meta-analyses^2,3^. The value of these findings, beyond that of scientific interest into how clinical features reflect treatment susceptibility, is also practical. Our results suggest ways to design clinical trials with greater power. Another important but unexpected finding is evidence of implicit selection bias in Crohn’s RCTs. Some degree of selection bias is to be expected of all trials insofar as they contain additional inclusion and exclusion criteria that are not a requirement for receiving clinical care. Indeed, we and others have observed this in the context of comparing RCT- and real-world cohorts^22–24^. What has been unclear, however, is the extent to which these biases may distort treatment outcomes. We identified a subgroup of anti-IL-12/23-preferring patients, mostly women over 50, that represented a miniscule fraction of trial subjects (2%). Yet, the typical prevalence of these patients as seen across 6 medical centers at the University of California suggests that as many as 25% of patients fall into this demographic. Of course, gender, older age, and race are not explicit exclusionary criteria in these registrational trials. Thus, it appears that these patients are systematically being under-enrolled. Future studies are needed to determine if this is the result of patient preferences, provider biases, or other factors.

To make our findings more accessible and to solicit early user feedback, we have prototyped a clinical decision support tool (crohnsrx.org). This tool is recommended for patients who generally resemble the subjects in the corresponding trials, particularly in terms of inclusion and exclusion criteria. Prospective validation of this decision support tool is forthcoming.

Of note, while this tool predicts treatment outcomes for the three drug classes modeled here, it cannot be used to estimate the causal effects of using concomitant immunomodulators or steroids. This was due to insufficient data on combination therapies to inform our models, with only 169 subjects in SONIC randomized to the combination of infliximab and azathioprine. Many trials contained patients who were already on immunomodulators at baseline, presumably reflecting a clinical history that predated their participation (e.g., severity of disease course). Given the lack of historical data that could explain these choices, this potential for confounding bias could not be analytically addressed here. Future work is needed to address these questions of combination therapies, incorporate data from trials of newer drugs, and further develop our prototype into an EHR-embedded tool that supports seamless, timely, and trustworthy recommendations at the point of care.

Strengths of this work include the strength and quality of the underlying data, the use of multiple methods (e.g., SRS, statistical hypothesis testing, cross-validation) to reduce bias, and several findings that advance the study of personalized medicine.

We acknowledge several limitations. This was a post-hoc analysis of randomized trials, and we cannot completely exclude residual biases. Prospective studies may increase confidence in these findings, particularly given apparent selection biases that could affect the application of trial-based insights to practice. There were several variables that we wanted to include in our models such as primary vs secondary loss of response to anti-TNFs, race/ethnicity, and comorbidities. Unfortunately, these data were not well-captured across the trials. We used linear models for this study, given their strengths (interpretability, inference, computational efficiency) as well as our findings that they produce similar results to random forests that model interactions. However, future studies using larger datasets and more model classes could revisit this choice, given the potential to discover new interactions with scientific and clinical importance. Our models collapsed multiple drugs into single drug classes to maximize statistical power. For example, we included certolizumab-related data to inform the anti-TNF drug class model, even though guidelines suggest that it may not be as effective as other TNFs^25^. Of note though, prior goodness of fit testing of drug-level models versus that of drug-class models showed no significant differences^4^. We were unable to include recently approved therapies due to restrictions on data access from trial sponsors, and this study was unable to assess combination treatments or inform treatment sequencing beyond the induction phase. Lastly, this study provides the most direct evidence for real-world patients who resemble trial subjects, both in explicit and implicit eligibility criteria. Future work is needed to confirm and extend these results to broader patient populations.

In conclusion, we performed an IPD meta-analysis of RCTs in Crohn’s disease. We identified multiple subgroups with different preferential responses to different drug classes, including one subgroup of women over 50 who may respond favorably to anti-IL-12/23 s after a trial of anti-TNFs. We uncovered potential evidence of selection bias in clinical trials and suggested ways to improve the efficiency and equity of these gold-standard studies. Lastly, we developed a prototype decision support tool to help improve treatment selection and patient outcomes for Crohn’s disease. Overall, we hope that this work will inspire additional investigations of personalized medicine across a wide range of diseases, utilizing the power of patient-level data captured over decades of clinical trials.

## Methods

### Ethics

This study was approved by the University of California, San Francisco Institutional Review Board (IRB). The need for informed consent was waived by the IRB as this study used deidentified data alone. The study and associated protocol were pre-registered on PROSPERO (#157827).

### Data access

In June 2019 we performed a search of clinicaltrials.gov to identify candidate studies to include in this planned meta-analysis. We identified 90 studies that were annotated as being completed, phase 2-4, randomized, double-blinded, interventional trials of treatments for Crohn’s disease at the FDA-approved route, dose, and frequency. We manually confirmed 16 trials as meeting these criteria^6–19^. To ensure comparability of the included cohorts and outcomes, we reviewed the major inclusion and exclusion criteria of all studies and confirmed that the Crohn’s Disease Activity Index (CDAI) had been captured at week six relative to treatment initiation. We also used the Cochrane Risk of Bias 2 tool to ensure that all included studies were at a low risk of bias (Supplementary Information). Following inquiries with the sponsors of these trials, we successfully obtained access to the IPD for 15 studies (N = 5703). These studies were conducted between 1999 and 2015 and corresponded to all six FDA-approved biologics as of 2019. All sponsors and data sharing partners agreed to place their data on a common, secure computing platform (Vivli) to facilitate downstream analysis.

### Quality control, harmonization, missing data

We performed extensive quality control evaluations of the included trials and data. This included confirming our ability to reproduce published statistics on the trial cohorts at baseline as well as the study primary endpoint. We were able to exactly reproduce most of the study results. Where discrepancies occurred, they were generally minor and fell within a 10% error bound. We reported major discrepancies to the study sponsor as per agreement. We attempted to completely eliminate all discrepancies, but this was not possible due to a variety of factors, including lack of access to the original analytic code or the complete analytic dataset, and inability to contact the original analysts.

We completed an assessment of data availability for all study variables. Target variables included demographic features, CDAI at baseline and week eight, baseline inflammatory biomarkers, concomitant steroid and immunomodulator use, history of treatment with anti-TNFs, and other disease-related features. We identified nine variables that were universally available across all trials and thus could be used for downstream modeling: Age, Sex, BMI, baseline CDAI, CRP, history of TNFi use, oral steroid use, immunomodulator use, and ileal involvement.

Only 3% of the participants had at least one missing covariate at baseline. Continuous variables were addressed by median imputation, and participants with missing categorical variables were dropped from the dataset (*N* = 86). 11% of the participants had a missing value for the outcome at week eight. To handle this, we used last-observation-carried-forward to impute these values, typically using measurements from week six and four. This is the typical practice for the analysis of these trials in regulatory submissions and was the prespecified approach in the protocols for all included trials. The variable corresponding to a history of TNFi use was available in all recent trials that occurred after the approval of the very first TNFi medication. Older trials of the first TNFis commonly excluded patients who had a history of exposure to other drugs from this class but did not include this feature as an actual variable in the data set. In these cases, we deterministically imputed this variable corresponding to no prior use.

Other variables of a priori importance could not be included in this study. Ethnicity was not collected in most trials. Race was missing in some trials, but when it was captured, it reflected a significant imbalance (88% of participants were white). Other disease-specific variables such as disease behavior and duration were also not uniformly captured across studies and thus could not be included in this meta-analysis.

The included trials had a range of study designs. We included both randomized and unblinded/open-label cohorts. For trials involving post-randomization gating (e.g., EXTEND, CLASSIC), we included those cohorts that were consistently exposed to a given treatment for six weeks only when post-randomization gating was not conditioned on treatment response (e.g., rerandomization of all participants, rather than just those with a particular response).

### Drug class modeling, subgroup identification

This study sequential regression and simulation (SRS) as the primary method to model potential outcomes under each drug class. SRS is described in detail and validated in a prior publication^4^. Briefly, SRS was developed to overcome several limitations of prior network meta-analyses (NMAs), which include their assumption of homogeneous cohorts, as well as the assumption that drug classes are just as likely to be tested against each other as they are against placebo. If all assumptions are met, NMAs can identify if some drugs are more effective or safe than others on average. However, they cannot be used to discover treatment subgroups, or identify if some patients would preferentially respond to one drug over another. This is because individual-level data are typically not available for these analyses.

Conventional individual participant data meta-analyses are the primary alternative to NMAs for comparative effectiveness studies. These use participant-level data, as does SRS, but historically have not been used to study heterogeneity in treatment effect. SRS was developed to normalize potentially heterogeneous trials with different placebo effects and enable the user to perform in silico head-to-head trials.

We used SRS to 1) normalize all trials to a common background (placebo response), and 2) analytically isolate the portion of the patient response that could specifically be attributed to a given treatment, rather than what would have been observed without treatment (i.e., placebo; Fig. 1b). For each drug class, we fit a separate linear mixed effects regression model of the drug-attributable reduction in CDAI. We chose to use linear models given the advantages in speed and interpretability, as well as evidence that they perform comparably to more flexible machine learning models (see Supplementary Table 2 of Rudrapatna et al. ^4^). This outcome was modeled as a function of the nine primary variables (see the “Quality Control” section above) handled as fixed effects, with trial as a random effect to control unmeasured heterogeneity across trials. We compared these models to intercept-only models using the likelihood ratio test. The latter corresponds to a model that ignores the role of patient-level characteristics in determining response to treatment and reflects the assumptions of methods that compare drugs based on their average effects, such as network meta-analyses. We performed Wald tests to identify significant predictors of responses to individual drug classes.

We applied the three finalized model objects to the covariate vectors of each of the 5703 participants in our meta-analysis to obtain their simulated response under each of these three counterfactual scenarios: treatment with an anti-TNF vs anti-integrin vs anti-IL-12/23. The inferred normal distributions of the conditional mean response to each drug class were pairwise compared against each other using the median of bootstrapped predictions and bootstrapped standard errors. We applied a nominal *p*-value threshold of 0.05 to identify patients belonging to a particular subgroup, defined as having a distinct pattern of ordinal preferences across all three drug classes. These included superiority of one drug class to another as well as indifference (lack of evidence for a difference at the *p* = 0.05 threshold).

Because the primary focus of this study involved the testing of only three primary hypotheses (i.e., goodness of fit for each of the drug class regression models compared to intercept-only models), we used nominal *p*-value thresholds of 0.05 for all other hypothesis tests including the post-hoc assessments of drug subgroup membership.

We note that SRS is associated with certain assumptions. These include 1) conditional exchageability of trial of origin in the placebo model (i.e., given the available variables used to model the placebo effect, the responses of placebo recipients are otherwise indistinguishable across trials, and 2) correct model specification (i.e., the functional form is correct).

### Subgroup assignment

For each trial-based patient (*N* = 5703) we predicted each drug class efficacy using the drug class models (Table 2; random effects set to 0) and estimated the 95% prediction interval using bootstrapping, the gold standard approach for deriving prediction uncertainty from linear mixed models^26^. We performed 10,000 simulations per patient. We conducted paired sample t-tests (*p* < 0.05) to further determine if any two drug class pairs were equivalent or different in efficacy to obtain a personalized treatment recommendation (Table 3). Finally, patients were assigned a subgroup based on their personalized treatment outcome based on the rank order and drug class comparisons.

### Subgroup validation via cross-validation

To further assess the validity of the subgroups identified by statistical hypothesis testing and reduce the risk of false subgroup discovery due to overfitting, we repeated the subgroup discovery procedure using tenfold cross-validation. We reserved 90% of the dataset to estimate placebo and drug class models, and we applied them to hold-out data from the remaining 10%. The conditional means and standard errors were used to perform pairwise t-testing and establish a rank-ordering of treatment preferences at the individual patient level.

### Decision support tool prototype

The decision support tool has been developed to provide real-time feedback to clinicians selecting treatments for patients with moderate-to-severe Crohn’s disease. However, we are also making a prototype of the tool publicly available to enable early feedback from many potential users and to provide insights to patients wishing to understand the potential advantages and disadvantages of available treatment options.

To use the decision support tool, users must input various data points, including the patient’s age, gender, BMI, recent c-reactive protein levels (measured in milligrams per liter), current corticosteroid and immunomodulator use (yes/no), prior anti-tumor necrosis factor use (yes/no), ileal involvement (yes/no), and the CDAI score. All inputs, except for the CDAI score, are mandatory for the calculation process. If any inputs are left blank, the user will receive an error message (Fig. 3a) and be prompted to input a default of “0” for numeric inputs or “No” for binary inputs if unknown. If the CDAI is unknown, the user can either 1) leave it blank, which will result in the tool imputing a score of 300 (indicative of moderate-to-severe disease), or 2) use the MDCalc CDAI calculator^27^ to obtain a precise result. If the CRP is unknown, it can be left blank and will be imputed to the median of the pooled population used for this study.

If all inputs are valid, the dashboard will output the patient’s treatment recommendations in both textual and graphical forms (Fig. 3b). To achieve faster recommendations in a real-time context compared to what would otherwise be obtained using bootstrapping, we used an analytical approximation for the standard error of a new prediction^28^. We used these standard errors to perform t-tests of the predicted mean response at week 6 for each pair of drug classes.

### University of California Health Data Warehouse

The University of California (UC) Health Data Warehouse (UCHDW) contains data on 8.7 million patients who have been seen at a UC facility since 2012; data has been stored using the Observational Medical Outcomes Partnership (OMOP) data model. Additional information about the OMOP common data model can be found at https://www.ohdsi.org/data-standardization/.

We queried the UCHDW to approximate a real-world subpopulation with similar characteristics to that of the anti-IL-12/23 subgroup found in our analysis, which consists of primarily older (>50 years old) and female participants. Queries were run on April 5th, 2023. We filtered patients in the UCHDW based on diagnoses (Crohn’s disease), medication prescriptions (adalimumab, ustekinumab, infliximab, natalizumab, vedolizumab, certolizumab pegol), medication start date, current age (as of 2023), and gender (Supplementary Information). We identified standard concept IDs for diagnoses and medications using the SNOMED International SNOMED CT Browser, Athena^29^. The codes are listed here: Crohn’s disease (201606), adalimumab (1119119), ustekinumab (40161532), infliximab (937368), natalizumab (735843), vedolizumab (45774639), and certolizumab pegol (912263). For more details on the query, please find the code at https://github.com/rwelab/CrohnsRx.

### Sample size calculations

We performed simulations to calculate the expected power of a prospective trial designed to test a key prediction of our model, that anti-IL-12/23 drugs are superior to anti-TNF drugs in women over 50. In each of 1000 simulations, we sampled from the overall trial population to create pairs of study arms consisting of women over 50. Sampling was done with replacement. We used the placebo and drug models to calculate the individual-level probability of achieving a CDAI reduction of ≥ 100 (i.e., clinical response), under an assumption of conditional normality. These were averaged within each simulated study arm, used to calculate the expected number of participants in clinical response, and then compared using a chi-squared test with an alpha of 0.05. This overall simulation procedure was performed using study arm pairs of sizes 100, 250, and 500. We repeated this analysis with the simpler inclusion criteria of just requiring participants to be over age 50, irrespective of gender.

### Statistical computing, web application development

Programming was performed in R (version 4.2.2). We used *RShiny* to prototype a decision support tool implementing our models (https://crohnsrx.org). The analytical code was reviewed by a second member of the team and has been placed on GitHub (https://github.com/rwelab/CrohnsRx). The web dashboard utilizes manually inputted data to produce recommendations based on our models. However, for users seeking to deploy this dashboard locally, an additional mode has been made available that automatically sources input data from an OMOP-formatted EHR database.

### Sensitivity analyses

We repeated the entire workflow of the study using random forest models. This involved the creation of four models: one for the placebo model and three for the drug class models. Each model was hyperparameter tuned by fivefold cross-validation. For subgroup assignment, we used bootstrap resampling to simulate mean potential outcomes from each drug class, and used the standard deviations of these bell-shaped distributions to perform pairwise t-testing as before.

After all subjects had been assigned to subgroups using the random forest-based approach, we calculated the proportion of patients who were placed in a subgroup that contradicted the subgroup assignments of the original linear mixed effects model. By contradictory, we mean a situation where drug class A was found superior to B in one modeling approach, but B was found superior to A in the other approach. Situations where A was superior to B in one approach but were considered statistically indistinguishable in the other were not counted as contradictory, but instead reflecting a lack of statistical power to resolve potential true differences in effects. We report the concordance as 1 minus the proportion of patients with contradictory assignments.

For each ablation analysis, we removed the target variable from the dataset and repeated the full procedure, then similarly calculated the concordance as above. We selected three target variables for this analysis: baseline CDAI, CRP, and ileal involvement

## Supplementary information

Supplementary information

## Data Availability

The raw data are owned by the trial sponsors. The data may be accessed for reproduction and extension of this work following an application on the YODA and Vivli platforms and execution of a data use agreement. However, a synthetic dataset needed to interpret, replicate, and extend this work is available at https://github.com/rwelab/CrohnsRx/.

## Code availability

The analytical code is freely available without restrictions at https://github.com/rwelab/CrohnsRx. This code, written in R (version 4.2.2) can be used together with the synthetic dataset to understand and extend the approach reported here.
